# Supplementary material for: Uremic Toxins and Ciprofloxacin Affect Human Tenocytes In Vitro
Source: Int J Mol Sci. 2020 Jun 14;21(12):4241. doi: 10.3390/ijms21124241 (PMC7353042; doi:10.3390/ijms21124241)
Supplement: Supplementary file 1 [file ijms-21-04241-s001.pdf]

Suppl. figures

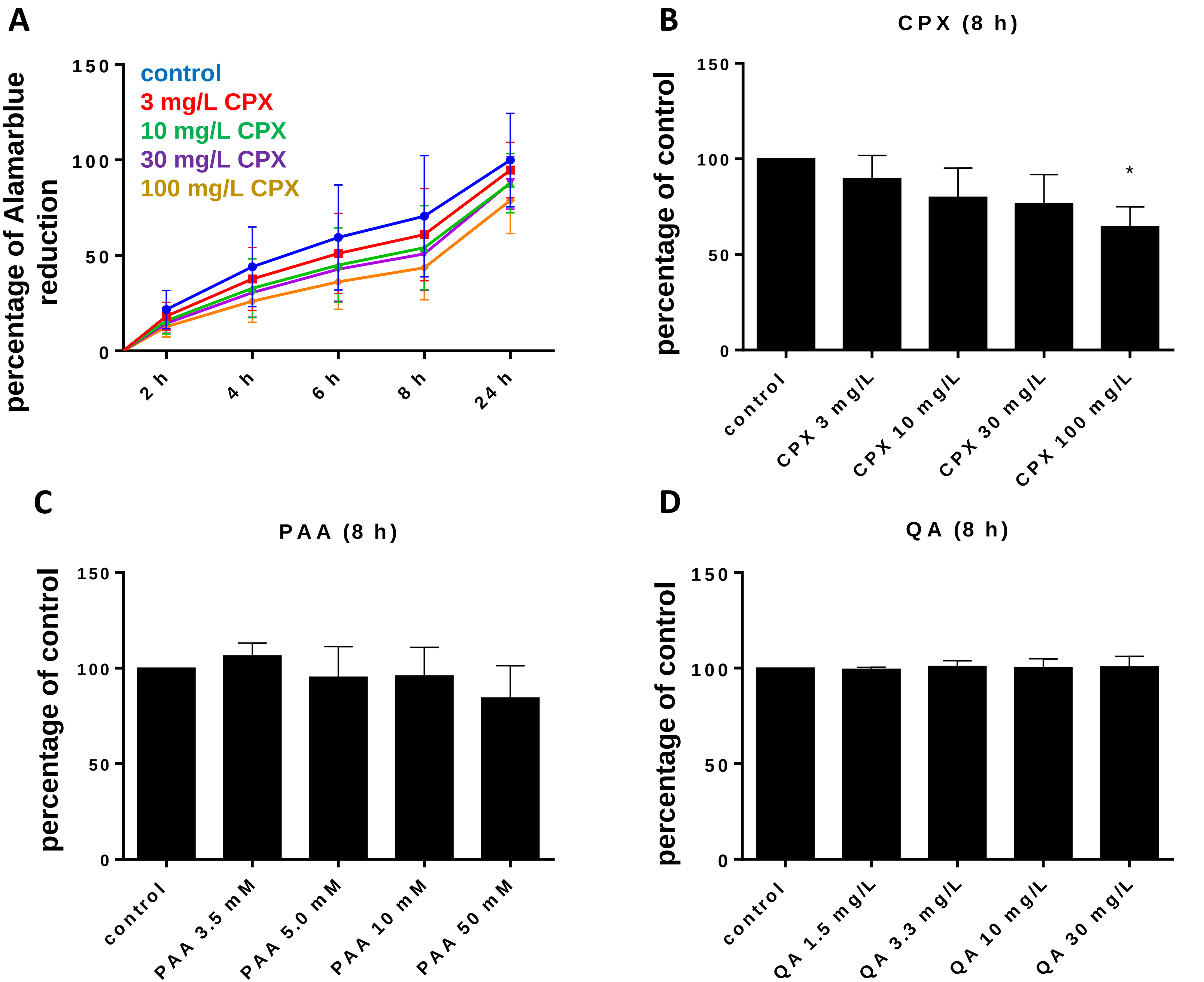
A
B
C
D
control
3 mg/L CPX
10 mg/L CPX
30 mg/L CPX
100 mg/L CPX
percentage of Alamarblue reduction
2 h
4 h
6 h
8 h
24 h
CPX (8 h)
percentage of control
CPX 3 mg/L
CPX 10 mg/L
CPX 30 mg/L
CPX 100 mg/L
*
PAA (8 h)
PAA 3.5 mM
PAA 5.0 mM
PAA 10 mM
PAA 50 mM
QA (8 h)
QA 1.5 mg/L
QA 3.3 mg/L
QA 10 mg/L
QA 30 mg/L
0
50
100
150

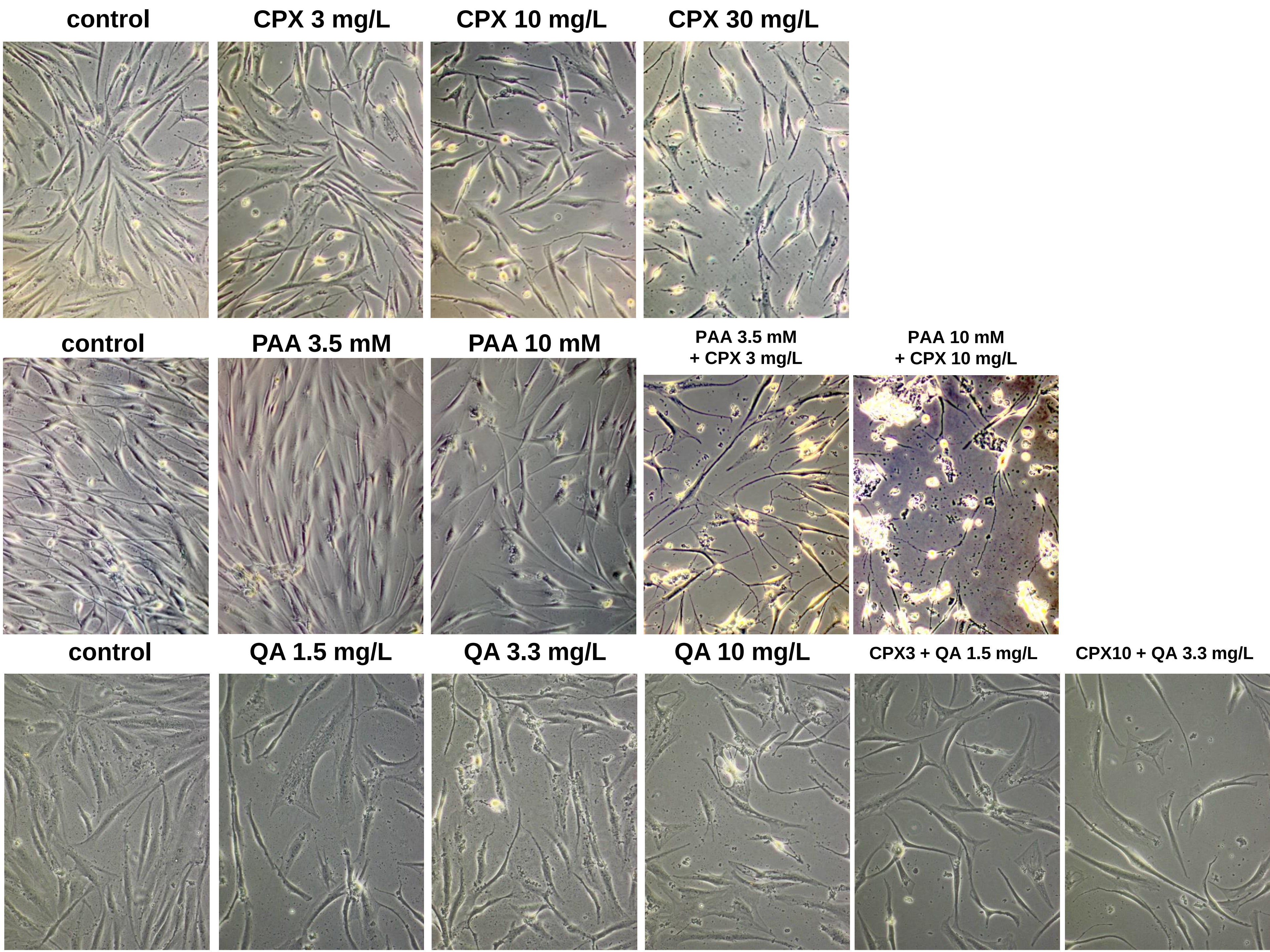
control
CPX 3 mg/L
CPX 10 mg/L
CPX 30 mg/L
control
PAA 3.5 mM
PAA 10 mM
PAA 3.5 mM
+ CPX 3 mg/L
PAA 10 mM
+ CPX 10 mg/L
control
QA 1.5 mg/L
QA 3.3 mg/L
QA 10 mg/L
CPX3 + QA 1.5 mg/L
CPX10 + QA 3.3 mg/L

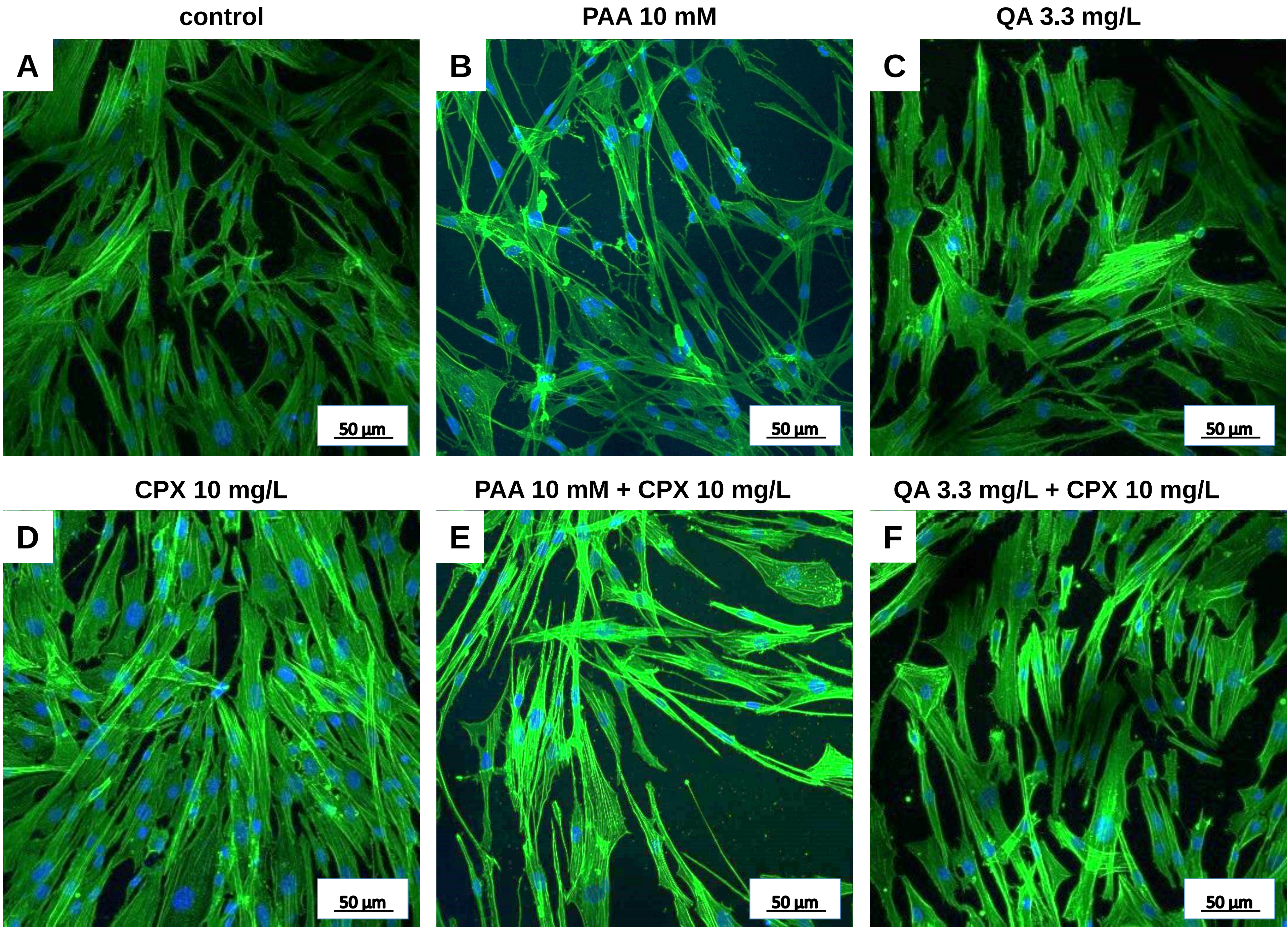
control
PAA 10 mM
QA 3.3 mg/L
CPX 10 mg/L
PAA 10 mM + CPX 10 mg/L
QA 3.3 mg/L + CPX 10 mg/L
A
B
C
D
E
F
50 µm
50 µm
50 µm
50 µm
50 µm
50 µm

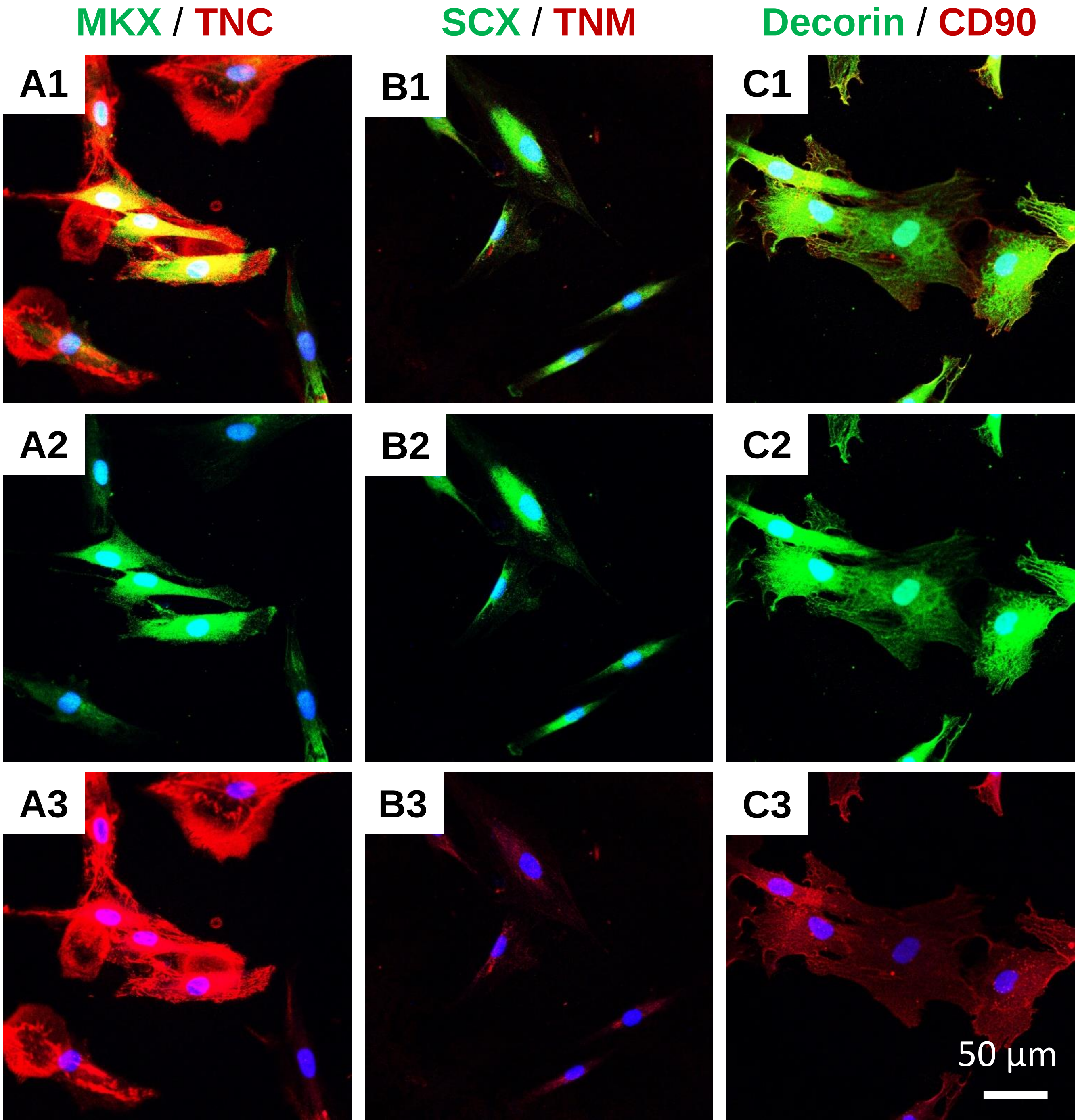
MKX / TNC
SCX / TNM
Decorin / CD90
A1
B1
C1
A2
B2
C2
A3
B3
C3
50 µm
